# An exploration of the relative importance of barriers hindering intensive combination treatment strategies in early rheumatoid arthritis

**DOI:** 10.1186/1472-6963-14-S2-P147

**Published:** 2014-07-07

**Authors:** Sabrina Meyfroidt, Marlies Hulscher, Diederik De Cock, Kristien Van der Elst, Johan Joly, René Westhovens, Patrick Verschueren

**Affiliations:** 1 Department of Development and Regeneration, KU Leuven, Leuven, Belgium; 2 Scientific Institute for Quality of Healthcare, Radboud University Medical Center, Nijmegen, The Netherlands; 3 Department of Public Health and Primary Care, KU Leuven, Leuven, Belgium; 4 Rheumatology, University Hospitals Leuven, Leuven, Belgium

## Background

The current recommendations for early Rheumatoid Arthritis (eRA) management focus on achieving clinical remission as soon as possible with an early and intensive treatment. Interventions tailored to prospectively identify barriers for the provision of early and intensive treatment are more likely to improve healthcare professionals’ adherence to treatment guidelines and to change practice in order to reflect current best evidence. The objective of this study was to explore the barriers and their relative importance for the provision of intensive combination treatment strategies (ICTS) in eRA from the healthcare professionals’ perspective.

## Materials and methods

Individual semi-structured interviews were conducted with 26 rheumatologists and 6 nurses participating in the CareRA trial. The CareRA trial is a Flemish multicentre RCT comparing different ICTS for eRA based on synthetic disease-modifying antirheumatic drugs in combination with a step-down glucocorticoid bridging scheme. Each interview was audio-taped, transcribed literally and thematically coded using the constant comparative method. The barriers identified from the interviews were incorporated in a Maximum Difference Scaling (MDS) survey, which was completed by 66 Flemish rheumatologists (response rate 44%). The MDS survey included 25 choice sets, each of which contained a different set of 4 barriers. In each choice situation, the rheumatologists were asked to select the most important barrier in their opinion. The mean relative importance score for each barrier was calculated using hierarchical Bayes modeling (Sawtooth Software’s SSI Web platform, version 8.2.0).

## Results

An initial list of 25 barriers emerged out of the interviews, including 10 treatment-, 4 healthcare professional-, 4 patient- and 7 environment-related barriers. The most important barriers identified from the MDS survey were: contraindication for some patients (e.g., patients with comorbidities, older patients); increased risk of side effects and related complications; and having to deal with patients’ resistance.

## Conclusions

Concerns regarding the suitability of ICTS for the individual patient and the complexity of prescribing a combination therapy including glucocorticoids were the most important barriers, highlighting the complexity of implementing ICTS for eRA in daily clinical practice. Implementation strategies for ICTS in daily practice should focus on physicians’ familiarity with the treatment strategies and patient education.

